# Resolving polymorphs and radiation-driven effects in microcrystals using fixed-target serial synchrotron crystallography

**DOI:** 10.1107/S2059798318010240

**Published:** 2018-11-09

**Authors:** Ali Ebrahim, Martin V. Appleby, Danny Axford, John Beale, Tadeo Moreno-Chicano, Darren A. Sherrell, Richard W. Strange, Michael A. Hough, Robin L. Owen

**Affiliations:** a School of Biological Sciences, University of Essex, Wivenhoe Park, Colchester CO4 3SQ, England; b Diamond Light Source, Harwell Science and Innovation Campus, Didcot OX11 0DE, England

**Keywords:** radiation damage, fixed-target serial crystallography, metalloproteins, polymorphism, room temperature

## Abstract

The ability to determine high-quality, artefact-free structures is a challenge in micro-crystallography, and the rapid onset of radiation damage and requirement for a high-brilliance X-ray beam mean that a multi-crystal approach is essential. However, the combination of crystal-to-crystal variation and X-ray-induced changes can make the formation of a final complete data set challenging; this is particularly true in the case of metalloproteins, where X-ray-induced changes occur rapidly and at the active site. An approach is described that allows the resolution, separation and structure determination of crystal polymorphs, and the tracking of radiation damage in microcrystals. Within the microcrystal population of copper nitrite reductase, two polymorphs with different unit-cell sizes were successfully separated to determine two independent structures, and an X-ray-driven change between these polymorphs was followed. This was achieved through the determination of multiple serial structures from microcrystals using a high-throughput high-speed fixed-target approach coupled with robust data processing.

## Abstract 2

Multiple dose-dependent room-temperature structures of copper nitrite reductase were determined from microcrystals in a single silicon nitride fixed-target chip. The separation of crystal polymorphs into distinct structures is described, together with the characterization of X-ray-irradiation effects through the dose series.

## 1. Introduction   

X-ray crystallography using synchrotron radiation is at the core of structural biology, providing atomic-level insight into key biological processes. However, it has long been recognized that the X-rays that are used to determine structures also cause changes to the crystal lattice and protein structure, a phenomenon known as radiation damage. Polymorphism and non-isomorphism of crystals have been a challenge in protein crystallography since the earliest days of the field, with an early example of non-isomorphism being the separation of lysozyme into type I and type II in the 1960s (described in detail in Blake *et al.*, 2012 ▸) to allow the structure determination of (type II) lysozyme. The rise of cryo-crystallography in the 1990s (reviewed by Garman, 1999 ▸) made single-crystal structure determination routine, but the use of more intense X-ray beams, and the desire to determine structures from ever smaller crystals, has made multi-crystal structure determination the norm once more.

Polymorphism can be present even between crystals harvested from the same crystallization drop, with variations in unit-cell parameters or even space group being observed. These differences may be owing to some external process such as heavy-atom derivatization, dehydration or cryocooling, where the resulting, unwanted, changes in cell dimensions can cause structure determination to fail (Crick & Magdoff, 1956 ▸). Differences can also arise from structural variation, where small changes in loops (Yogavel *et al.*, 2010 ▸) or conformational flexibility (Redinbo *et al.*, 1999 ▸) can result in significant changes in cell dimensions and space group. Non-isomorphism can also take more subtle forms deriving from, for example, weakly bound ligands, only becoming apparent in electron-density maps following careful cross-comparison of many data sets (Pearce *et al.*, 2017 ▸). In the case of multi-crystal and serial micro-crystallography data-set formation, however, larger scale differences are often used as the basis for the formation of a final data set through brute-force merging or a more refined approach such as hierarchical cluster analysis (Foadi *et al.*, 2013 ▸; Santoni *et al.*, 2017 ▸). Alternatively, statistical approaches such as the use of a genetic algorithm to optimize data-quality metrics such as the *R* value or 〈*I*/σ(*I*)〉 can also be used to obtain a single high-quality data set from many crystals (Zander *et al.*, 2016 ▸), although in the future this could also be used to identify and separate non-isomorphous groups.

Radiation damage results from energy deposited in crystals by X-rays and is manifested in two ways. Firstly, global radiation damage results in changes to the unit cell, increased disorder and a loss of diffracting power and consequently resolution [for comprehensive reviews of radiation damage in macromolecular crystallography, see Holton (2009 ▸) and Garman (2010 ▸)]. Secondly, site-specific radiation damage, which is most commonly observed in the form of disulfide reduction, the decarboxylation of side chains, and the reduction of metals and other redox centres. These changes occur on different dose scales and are temperature-dependent. Cryocooled (100 K) crystals are considered to no longer give useful diffraction beyond absorbed doses of 30 MGy (the Garman limit; Owen *et al.*, 2006 ▸). Despite the protection that cryocooling confers, site-specific changes occur at significantly lower doses than this, with the reduction of redox centres occurring at doses as low as 10 kGy in some cases (Kekilli *et al.*, 2017 ▸), some 3000 times lower than the Garman limit, a dose that can be achieved in a few milliseconds at modern synchrotron beamlines (Owen & Sherrell, 2016 ▸).

The vast majority of protein structures have been determined at 100 K in order to mitigate the global effects of radiation damage. The use of low temperatures, and an ‘as low a dose as practicable per data set’ strategy, mitigates damage and allows experiments such as multiple structures from one crystal (MSOX) to be performed in which electron-driven catalysis outruns electron-driven damage processes (Horrell *et al.*, 2016 ▸). At elevated temperatures both ‘normal’ data collection and experiments such as MSOX become considerably more challenging as the rates of damage increase.

The drawback of increased rates of damage must be weighed against the benefits that data collection at higher temperatures provides: decreased viscosity and increased thermal motion that allow more functionally relevant changes to be observed. Protein dynamics and reactivity are considerable within the crystal lattice, but are partly suppressed in the highly viscous, glassy solvent environment of crystals cooled to 100 K. Increases in mosaicity from cooling may be avoided by working at a temperature close to that at which the protein was crystallized. Increased reactivity within crystals, additional conformations of side chains and differences in ligand binding are observed when working at room temperature (RT; Fraser *et al.*, 2011 ▸; Fischer *et al.*, 2015 ▸), and there is a considerable incentive to determine structures at ‘close to physiological’ temperatures. RT structures may also be more directly relatable to solution kinetics experiments.

In terms of radiation damage, variable-temperature experiments have shown that most of the dose-lifetime extension gained at 100 K remains present at temperatures as high as 200 K (Warkentin *et al.*, 2012 ▸, 2013 ▸). Elevated-temperature crystallography remains a considerable challenge, however, particularly for small weakly diffracting crystals, which require a tightly focused intense beam. Despite these experimental challenges, MSOX series have been successfully determined at 190 K (where much of the advantage for crystal lifetime of data collection at 100 K is maintained but dynamic freedom is increased) and at RT using macrocrystals (Horrell *et al.*, 2018 ▸), revealing the considerable benefit of working at higher temperatures in that more of the reaction may be observed in the lifetime of the crystal owing to the higher dynamic freedom of the crystalline enzyme.

Here, we describe the development of a modified MSOX approach applied to microcrystals at RT. This approach is no longer multiple structures from one crystal, but multiple serial structures from many crystals (MSS). In order to effectively produce MSS series from microcrystals there are significant technical and methodological challenges to overcome: approaches for serial crystallography at synchrotron and XFELs provide a way forward.

Serial synchrotron crystallography (SSX) is an emerging area allowing structure determination by recording single images from many thousands of crystals (Diederichs & Wang, 2017 ▸). Typically, the data obtained are merged to provide a single structure. A number of sample-delivery methods to realize SSX have been developed, but a particular advantage of fixed-target approaches is that they can be readily modified to facilitate the determination of multiple structures.

MSS experiments require the ability to record data from a large number of crystals at a large number of dose points. The dose is controlled either by varying the beam intensity or the time that the crystal spends in the beam, and should be as similar as possible for all of the crystals studied, for example by matching aperture and crystal sizes and using a sample in which the microcrystals are relatively homogeneous in size and morphology. Other relevant factors include accurate and consistent stage movement and exposure timing. In order to follow a reaction, a number of well spaced dose points are required. In this work, we have examined the feasibility of measuring multiple data sets in close succession from microcrystals using fixed-target SSX. Using this approach, tens of dose-dependent data sets may be obtained highly efficiently, with each microcrystal exposed for a total of only a few hundred milliseconds.

We describe the approach with a detailed case study of MSS experiments on microcrystals of copper nitrite reductase from *Achromobacter cycloclastes* (*Ac*NiR). The nature of the data gained and the global and site-specific radiation-induced changes to the structure are presented. We also describe a practical approach to separating polymorphs within a population of microcrystals and obtaining separate structures of each form, leading to two separate MSS series collected from a single batch of crystals.

## 2. Materials and methods   

### 2.1. Crystallization and crystal loading to fixed targets   

Recombinant *Ac*NiR was expressed and purified as described previously (Horrell *et al.*, 2016 ▸). Batch microcrystals were prepared by rapidly mixing 20 mg ml^−1^ *Ac*NiR in 20 m*M* Tris pH 7.5 with a solution consisting of 2.5 *M* ammonium sulfate, 0.1 *M* sodium citrate pH 4.5 buffer in a ratio of 1:3 and mixing by vortexing for 60 s. Microcrystals with a diameter of 5–15 µm grew at room temperature over a period of 4–6 d. Microcrystal suspensions were centrifuged at 800 rev min^−1^ for 30 s to sediment the crystals; the crystallization buffer was then removed and replaced with a storage buffer consisting of 1.6 *M* ammonium sulfate, 0.1 *M* sodium citrate pH 4.5. Crystals were soaked in a solution of mother liquor supplemented with 100 m*M* sodium nitrite for a duration of 20 min prior to loading onto the chip. Serial dilutions were achieved by adding additional storage-buffer solution.

**Figure 1:**
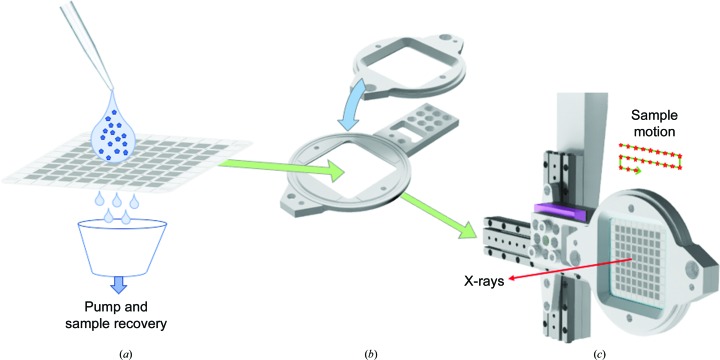
Schematic of chip-loading procedure. (*a*) The microcrystal suspension is pipetted onto the surface of the glow-discharged chip, with excess liquid being removed by the application of suction to the opposite surface. (*b*) Placing chips into the holder: a thin film of Mylar held in place by O-rings seals the chip and prevents drying out. (*c*) Loading of the chip and holder assembly onto the beamline sample stage during sample exchange: a kinematic mount (magenta) holds the chip in place in a precise and reproducible position, with subsequent alignment carried out using fiducial markings on each chip. The direction of the X-ray beam is indicated as a red arrow, while a schematic of chip movement is shown in green and red stars indicate positions where the chip is stationary and data are collected.

Silicon nitride fixed targets or ‘chips’ of a new design, but based on those described previously (Mueller *et al.*, 2015 ▸; Oghbaey *et al.*, 2016 ▸), were used for the experiments described here. These chips follow the funnel-like design of previous chips but utilize a new higher capacity layout while retaining approximately the same dimensions (30 × 30 mm). Each chip comprises 8 × 8 individual ‘city blocks’, with each city block containing 20 × 20 apertures; the nominal capacity of each chip is therefore 25 600 (Fig. 1 ▸). The apertures are funnel-shaped, with the smaller end being 7 × 7 µm and the larger end being 85 × 85 µm.

Chips were prepared by glow-discharge cleaning in a similar manner to the cleaning of cryo-EM grids. Glow discharging improved the dispersion of the crystal slurry on the chip surface, reducing the volume of crystal slurry required to load a chip, and also resulted in improved drawing of liquid through the chip apertures. Chips were loaded within a humidity-control enclosure (Solo Containment, Cheshire, England). Typically, 100–200 µl of a microcrystal suspension was pipetted onto the surface of the chip, after which gentle suction was applied from below to draw microcrystals into the individual wells. The chip was then sealed between two layers of 6 µm thick Mylar in the sample holder before transfer to the beamline (Fig. 1 ▸).

### 2.2. Moving chips/instrumentation and beamline parameters   

Instrumentation for the movement of chips through the X-ray beam was mounted on beamline I24 at Diamond Light Source as described previously (Owen *et al.*, 2017 ▸). An X-ray beam size of 8 × 8 µm (full width at half maximum; FWHM) was used. All data were measured at 12.8 keV using a PILATUS3 6M detector with a crystal-to-detector distance of 310 mm. The beam flux of 3.0 × 10^12^ photons s^−1^ was measured immediately prior to the experiments using a silicon PIN diode as described previously (Owen *et al.*, 2009 ▸) and was attenuated tenfold for the data collections described below. The dose absorbed by each crystal was estimated using *RADDOSE*-3*D * (Zeldin, Gerstel *et al.*, 2013 ▸). Note that for the beam parameters described here, a diffraction-weighted dose (Zeldin, Brockhauser *et al.*, 2013 ▸) of 11 kGy corresponds to a maximum dose (as reported by older versions of *RADDOSE*) of 31 kGy. Data-collection parameters are shown in Table 1 ▸. As with all serial experiments, there will be some crystal-to-crystal variation in absorbed dose and so the calculated dose represents an average value. The largest difference in absorbed dose is likely to arise in crystals which are only partially exposed to X-rays if, for example, they are not centred in the apertures of the chips. We sought to minimize the variation in absorbed dose by ensuring that the beam size and intensity remained unchanged over the duration of the experiment, matching the beam size and chip aperture, and using crystals of a single morphology and of similar size.

### 2.3. Data-collection strategies   

**Figure 2:**
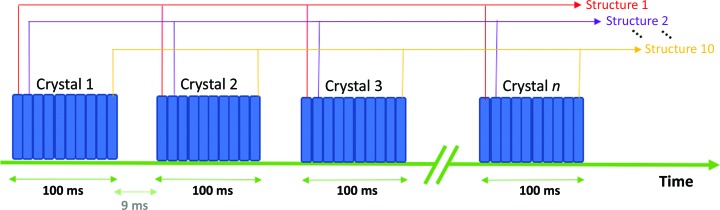
Schematic of MSS data collection and formation of dose series. At each position of the fixed target, multiple images are measured in shutterless mode using the PILATUS3 6M. In the example shown here, ten sequential exposures of 10 ms are recorded at each position and the translation time between positions is 9 ms. Images from each dose series are then grouped together in time (dose) bins, allowing dose-dependent structures to be obtained.

Rapid data series were measured from each aperture of a chip, with up to 20 diffraction images recorded at each position prior to translation to a fresh aperture. Data from each short series could then be sorted into dose bins and selectively merged, allowing dose-dependent structures to be obtained. This movement and dose-binning strategy is shown schem­atically in Fig. 2 ▸. Typically, an exposure period of 10 ms is used for each image, meaning that a ten-frame series (100 ms X-ray exposure per crystal) can be recorded from an entire chip (25 600 positions) in 46 min. The image series at each position was individually triggered using a Keysight 33500B signal generator, which in turn was triggered by a DeltaTau Geobrick LV-IMS-II stage controller when each crystal position had been reached. The X-ray shutter remained open for the duration of data collection and was not closed between apertures on a chip.

### 2.4. Data processing, structure solution and refinement   

Data took the form of sequentially numbered images in CBF format. All images were indexed using *dials.still_process* in *DIALS* v.1.8.5 (Winter *et al.*, 2018 ▸) with subsequent scaling and merging performed using *prime* (Uervirojnangkoorn *et al.*, 2015 ▸). As an example of typical data volumes, throughputs and hit rates, the set of 20 dose points described below comprised some 500 000 images collected in less than 3 h. Bragg peaks were observed on 332 272 images, and 589 403 patterns were indexed (owing to multi-lattice indexing of up to three patterns per image). Of the indexed patterns, the percentages with single, double and triple lattices were 36, 36 and 28%, respectively.

Data were binned into the different dose points to produce final MTZ files for each MSS data set. The indexing ambiguity in space group *P*2_1_3 was resolved by the use of a reference data set collected from a single *Ac*NiR crystal at 100 K. Owing to the manner in which serial crystallography data are collected, ‘traditional’ metrics such as *R*_merge_ which compare individual measurements do not reflect the quality of the data set. The quantities *R*_split_ (White *et al.*, 2016 ▸), which compares separately merged halves of the data, and CC_1/2_ (Karplus & Diederichs, 2012 ▸), which reports on the precision of merged measurements, were therefore used to assess the quality and resolution of each data set (Table 1 ▸). Both the mean intensity 〈*I*〉 and signal-to-noise ratio *I*/σ(*I*) of each data set are also quoted; comparatively low values of *I*/σ(*I*) reflect the challenge of accurately estimating the error of intensities in monochromatic serial data.

Structures were refined from a starting model of the room-temperature *Ac*NiR–nitrite complex, from which water and ligands had been removed (PDB entry 5i6l; Horrell *et al.*, 2016 ▸), using *REFMAC*5 (Murshudov *et al.*, 2011 ▸) in the *CCP*4*i* interface and/or *PHENIX* (Adams *et al.*, 2010 ▸). Structures were rebuilt in *Coot* (Emsley *et al.*, 2010 ▸) between rounds of refinement, and validation was performed using tools within *Coot*, *MolProbity* (Chen *et al.*, 2010 ▸), the *JCSG Quality Control Check* server (https://smb.slac.stanford.edu/jcsg/QC/) and the PDB validation server (https://validate-rcsb-1.wwpdb.org). Side-chain atoms that were not supported by electron density were deleted from the model. Coordinates and structure factors were deposited in the RCSB Protein Data Bank with the accession numbers given in Table 1 ▸. Surface areas and volumes were calculated in the 3*V* volume assessor (Voss & Gerstein, 2010 ▸).

## 3. Results and discussion   

### 3.1. Discrimination between *Ac*NiR crystal polymorphs in the microcrystal population   

**Figure 3:**
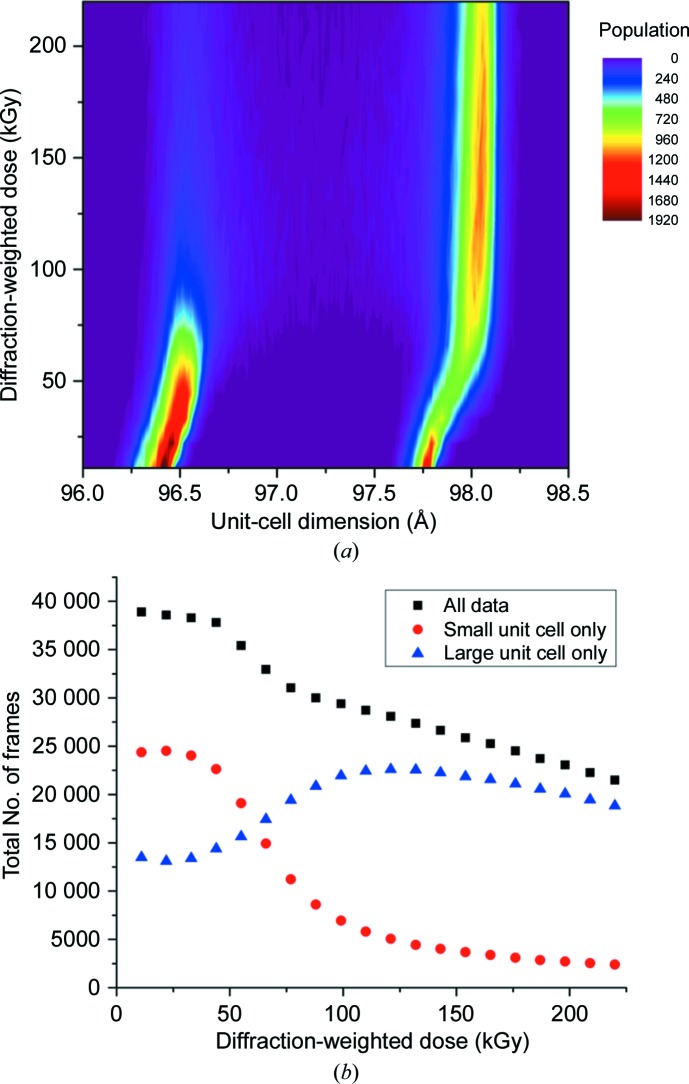
(*a*) Two-dimensional histogram showing changes in unit-cell dimension (*a* = *b* = *c* in space group *P*2_1_3) and population across a chip as a function of absorbed dose. Populations are the numbers of indexed images in unit-cell bins of width 0.01 Å. The starting unit-cell dimensions of the polymorphs are 96.38 and 97.75 Å; these increase to 96.56 and 98.04 Å at 220 kGy. (*b*) Number of integrated images of each polymorph on the chip as a function of absorbed dose. Images were selectively integrated into ‘small’ or ‘large’ unit-cell groups (details are given in the text).

**Figure 4:**
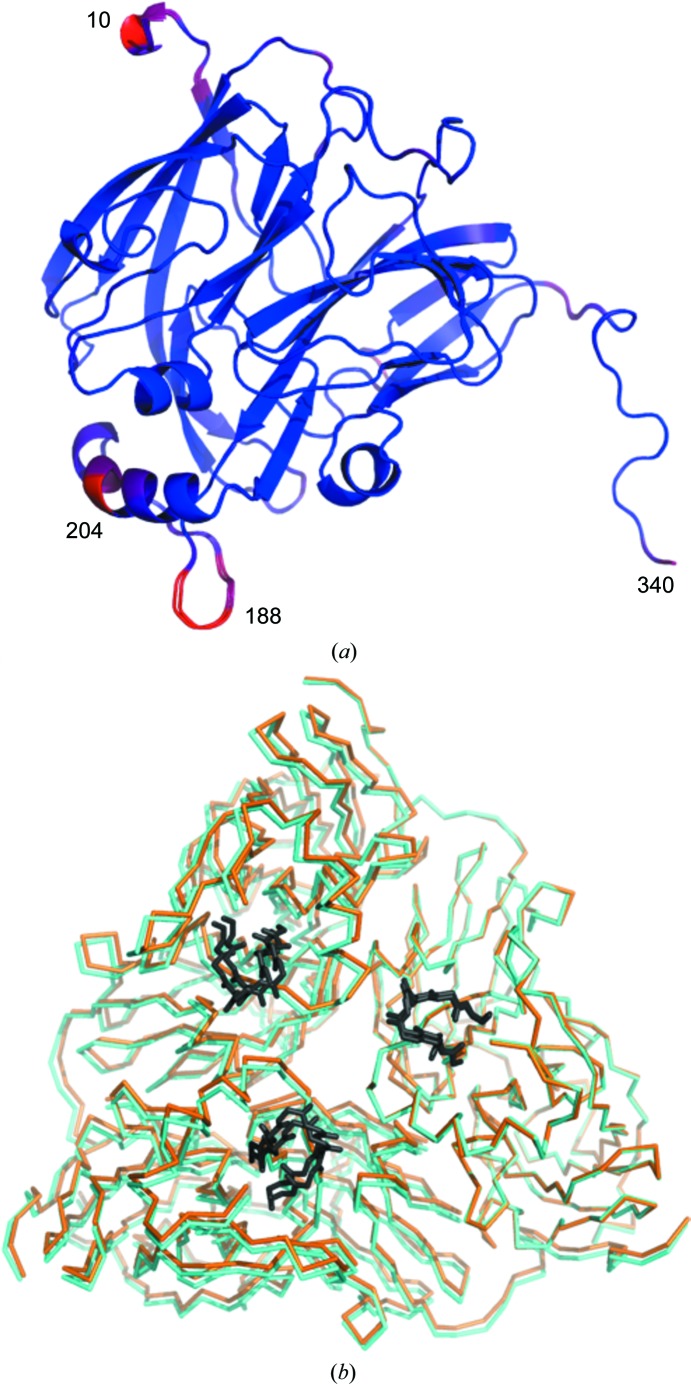
(*a*) Superposition of the data set 1 structures of *Ac*NiR-big and *Ac*NiR-small coloured by r.m.s.d. between the structures from low values (blue) to high values (red). (*b*) Superposition (using monomers *A*) of trimers generated by crystal symmetry for data set 1 of *Ac*NiR-small (orange) and *Ac*NiR-big (cyan). The view is down the threefold axis. While the structures of individual monomers are very similar, structural changes across the trimer are larger and are related to the change in unit-cell volume. The loop regions (residues 186–193) are shown in dark grey.

A total of 20 successive images of 20 ms exposure each were measured at each aperture position of a chip, providing 20 room-temperature data sets at different dose points. Indexing of individual diffraction patterns (stills) from the first image measured from each *Ac*NiR microcrystal revealed a bimodal distribution of unit-cell parameters (Fig. 3 ▸). As a consequence, images were binned into two groups above and below a mid-peak cutoff of 97.25 Å, which we henceforth refer to as *Ac*NiR-big (*a* = *b* = *c* = 97.75 Å) and *Ac*NiR-small (*a* = *b* = *c* = 96.38 Å). Data-processing statistics for each group (for data set 1) compared with those obtained by merging all patterns are given in Table 1 ▸. Separation of the unit-cell polymorphs led to an improvement in data quality, suggesting that this analysis step is beneficial over simply merging all data regardless of unit-cell parameter. Refinement of the structure arising from each polymorph revealed two different structures, as shown in Fig. 4 ▸. The *Ac*NiR-big and *Ac*NiR-small structures were superimposable with an r.m.s.d. of 0.16 Å (Supplementary Fig. S1). While the overall *Ac*NiR-big and *Ac*NiR-small structures were very similar, significant structural differences were observed at the N- and C-termini and in the loop structure around residues 187–193 and 201–205 (Fig. 4 ▸ *a*). Examination of the symmetry-generated *Ac*NiR trimer (the biological assembly) revealed further differences related to the unit-cell polymorphs (Fig. 4 ▸ *b*), whereby the volume of the *Ac*NiR-big trimer was 157 399 Å^3^, an increase of some 1901 Å^3^ over the *Ac*NiR-small volume of 155 498 Å^3^. The corresponding increase in surface area was 659 Å^2^: from 22 998 Å^2^ for *Ac*NiR-big to 22 339 Å^2^ for *Ac*NiR small.

**Figure 5:**
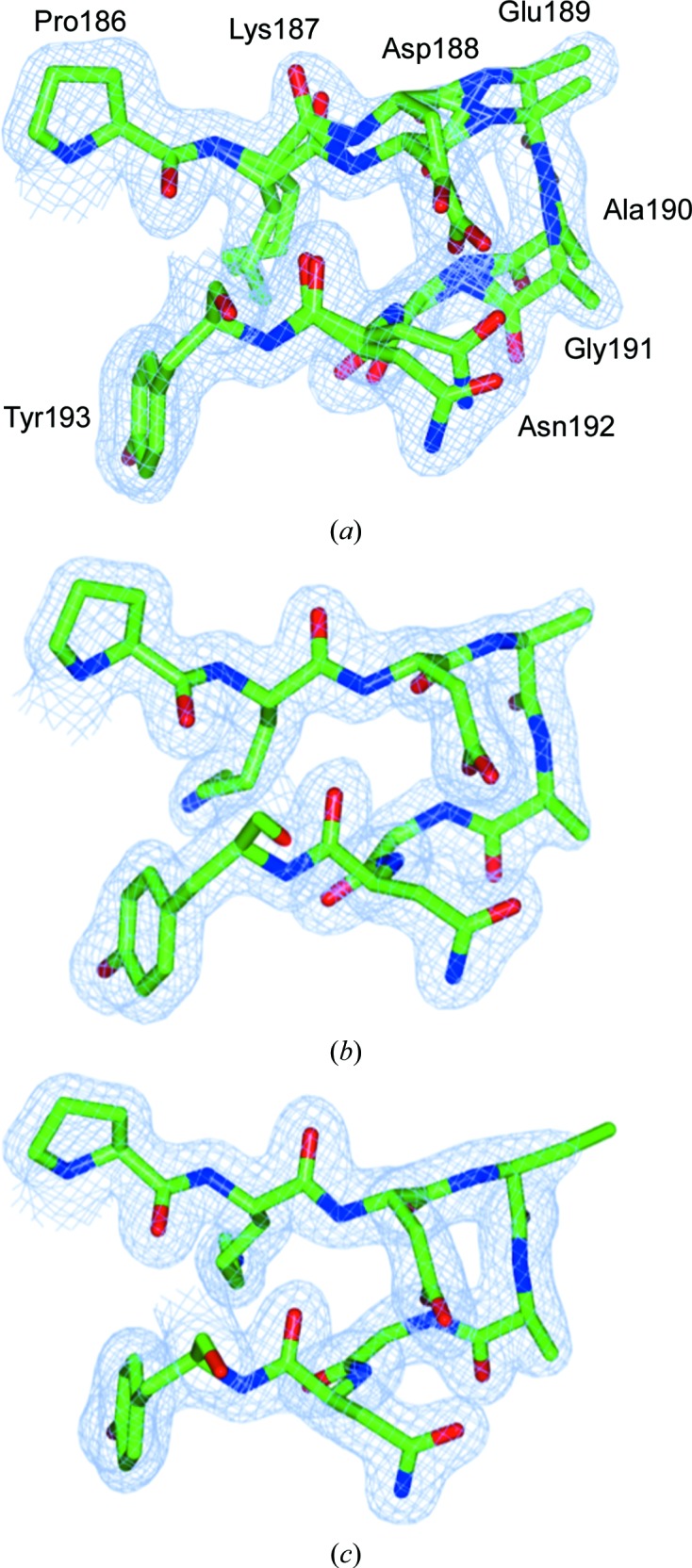
2*F*_o_ − *F*_c_ electron-density maps for the *Ac*NiR data set 1 structures derived from (*a*) all images, (*b*) the small unit cell only and (*c*) the large unit cell only. The maps are contoured at 0.311 e^−^ Å^−3^ (all data), 0.368 e^−^ Å^−3^ (*Ac*NiR-small) and 0.348 e^−^ Å^−3^ (*Ac*NiR-big). Note the dual conformations in (*a*) with occupancies (0.3 and 0.7) consistent with the proportion of large-cell and small-cell images within data set 1. This figure was prepared using *CCP*4*mg* (McNicholas *et al.*, 2011 ▸).

Without the polymorph-separation procedure, the single combined data set produced an electron-density map with dual conformations representing the two polymorph structures (Fig. 5 ▸). Comparison of these structures with earlier 100 K crystal structures (for example PDB entry 2bw4, where the unit-cell parameter was 95.41 Å; Antonyuk *et al.*, 2005 ▸) revealed a pattern of changes that affect the crystal contacts between *Ac*NiR monomers. These structural differences between the two polymorphs are explored further in §[Sec sec3.3]3.3.

The reason why two different polymorphs exist within a single sample of batch-grown *Ac*NiR microcrystals and the factors determining the proportion of each that is present remain unclear. Interestingly, different batch crystallizations of similar age exhibited different proportions of *Ac*NiR-big and *Ac*NiR-small (data not shown). To investigate possible causes of the polymorph distribution, a second chip of *Ac*NiR microcrystals was used in which different dilutions of the starting microcrystal stock were applied to different ‘city blocks’ of the chip. A progressive shift with dilution was observed, from an almost entirely small-cell population in the undiluted sample to a predominantly large-cell population at dilutions greater than 15× (Supplementary Fig. S2). The mechanism by which dilution influences the cell polymorph remains unclear. We note that as the dilution was achieved by the addition of further crystal-storage buffer to the microcrystal suspension, there were no changes to the pH or the precipitant concentration in this process.

### 3.2. Global radiation damage in MSS data sets   

**Figure 6:**
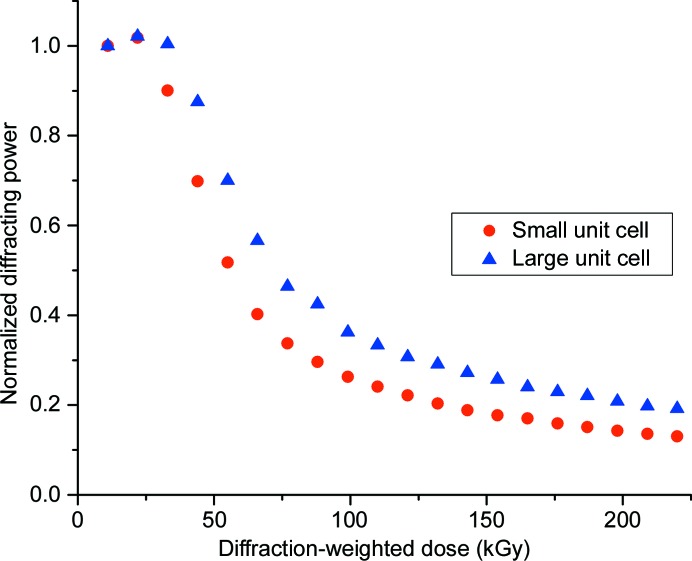
Total diffracting power of crystals as a function of accumulated X-ray dose. Diffracting power was defined as the total Bragg intensity for each dose point as reported by *prime* after integration and scaling over the resolution range 30–1.48 Å.

As dose was accumulated at a relatively high dose rate of 1.1 MGy s^−1^, evidence of global radiation damage was found in the form of a rapid decrease in diffracting power (Fig. 6 ▸). An initial plateau region or lag phase is apparent spanning the first three data sets, corresponding to ∼20–35 kGy and 20–30 ms exposure time. The subsequent fall in diffracting power follows an exponential decay, with a somewhat faster fall-off with dose for the *Ac*NiR-small population than the *Ac*NiR-big population. The half-doses for the two polymorphs were 55 and 75 kGy, respectively (as estimated from Fig. 6 ▸). The half-dose for the large-cell polymorph is larger than that for the small cell owing to the convolution of the decay in diffracting power with the radiation-driven polymorph switch (Fig. 3 ▸ *b*).

Such an intensity decay can arise from a number of sources. Firstly, exceeding the count-rate limit of a single photon-counting detector such as the PILATUS3 could result in a plateau. Care was taken to ensure that count rates were well below the maximum of 10 × 10^6^ counts per second per pixel that PILATUS3 detectors are capable of accurately recording. In the experiments described here the maximum observed counts in a Bragg spot was ∼8000, corresponding to a count-rate of 0.4 × 10^6^ counts per second per pixel or 4% of the maximum count rate. More typical maximum counts in a Bragg spot were <4000, or less than 2% of the maximum. Further, as the crystals are not rotated at all during data collection, count rates are likely to be steady throughout the duration over which an image is recorded: a key assumption in any count-rate correction that is made during detector readout. Secondly, a lag phase can result from the outrunning of global effects such as beam-induced heating. In an initial period when site-specific damage dominates, the global decay in intensity will deviate from an exponential decay (Sygusch & Allaire, 1988 ▸, Owen *et al.*, 2014 ▸). Thirdly, as the beam size is approximately equal to the aperture size, any temporal error in chip motion and detector triggering would mean that the diffracting power observed in the first image would be significantly reduced. Considering the horizontal beam profile, chip aperture and motion only, 68% of the beam intensity (beam FWHM 8 µm) falls within the 7 µm chip aperture assuming that they are perfectly co-centred. If the chip aperture is offset by 1, 2 or 3 µm the intensity incident on the aperture falls by 3, 11 or 23%, respectively. Thus, small errors in positioning can result in large differences in the recorded intensity. While extreme care was taken to tune the stages to eliminate this as a source of systematic error, it cannot be discounted. Fourthly, the Gaussian non-tophat profile of the beam can affect intensity decay. Warkentin and coworkers examined the effect of beam profile and dose rates on the rates of global radiation damage at room temperature in thaumatin and lysozyme crystals, showing that a non-uniform beam profile can result in a non-exponential dose response. All of these factors may contribute to the intensity-decay profile observed (Warkentin *et al.*, 2017 ▸).

Individual MSS data sets in the series of 20 showed good-quality merging statistics to a resolution of 1.48 Å (Table 1 ▸), despite a nominal resolution limit of 1.7 Å imposed by the crystal-to-detector distance (inscribed circle on the detector surface). This is owing to the extremely high redundancy achieved with our high hit-rate chip-based serial data collection. Note that in the case of small-cell *Ac*NiR, in addition to a global decrease in diffracting power, the decrease in resolution is significantly affected by the reduction in population resulting from X-ray-driven transfer from the small-cell to the large-cell *Ac*NiR polymorph (Table 1 ▸). The resulting decrease in multiplicity significantly impacts the resolution to which acceptable merging statistics are obtained.

### 3.3. Changes to polymorph populations with X-ray dose   

Monitoring of the mean cell dimension in each polymorph together with the population distribution reveals intriguing changes as X-ray dose is accumulated (Fig. 3 ▸). The mean cell dimension within each population undergoes a small, progressive increase (Supplementary Fig. S3) consistent with many previous studies showing unit-cell expansion with dose. While often observed, unit-cell expansion is generally not regarded as a reliable metric of radiation damage owing to the lack of reproducibility of the effect between different crystals of the same protein (Murray & Garman, 2002 ▸). Remarkably, however, in our MSS data an interchange between the unit-cell polymorph populations throughout the dose series is also evident (Fig. 3 ▸ *b*). The *Ac*NiR-small population rapidly decreases, with a concomitant increase in the population of *Ac*NiR-big. It is apparent from Fig. 3 ▸(*a*) that (i) the switch from small to large cell for any particular microcrystal yields a cell that is consistent with the ‘damaged large cell’ of a particular dose rather than the large cell at dose point 1 and (ii) the lack of overlap between the two populations implies a specific structural change between polymorphs. Interestingly, the increase in unit cell for both polymorphs begins immediately upon irradiation (Fig. 3 ▸, Supplementary Fig. S3), while the switching of polymorphs is minimal within the first 100 ms before proceeding rapidly. While not conclusive, this could suggest that expansion of the small cell acts as a trigger or seed leading to subsequent polymorph swapping.

As structural differences in the loop region around Asp188 were observed between polymorphs, further comparison was undertaken to seek to understand the mechanism behind the switching between populations. Multiple single-crystal structures are available for resting-state AcNiR, primarily measured at 100 K but also at elevated cryogenic temperatures (Sen *et al.*, 2017 ▸). Superposition of this loop region in *Ac*NiR structures determined at different temperatures (Supplementary Fig. S4) reveals a progressive shift in the position of the loop from 100 K (cell length 95.41 Å) to 240 K (PDB entry 5n8f; 96.13 Å) and RT (PDB entry 5off; 96.23 Å). The latter is very similar in structure to the *Ac*NiR-small polymorph (96.38 Å). A further shift then occurs to the *Ac*NiR-big polymorph (98.21 Å). While further work is required to prove a link, the apparent correlation of loop position with cell length suggests that this loop reorganization may indeed be related to the switching of polymorphs.

The precise mechanism by which the large unit-cell expansion from *Ac*NiR-small to *Ac*NiR-big occurs upon irradiation remains unclear, but insights may be gained from known site-specific radiation-damage phenomena. Decarboxylation of aspartic acid and glutamic acid residues has been well characterized (Burmeister, 2000 ▸; Holton, 2009 ▸; Garman, 2010 ▸), and notably the dual conformation of the loop region contains two such residues: Asp188 and Glu189. To examine this possible cause of the shift between polymorphs, the structure of data set 15 with the large-cell polymorph was examined (Table 1 ▸, Supplementary Fig. S5). The electron density for Asp188 remained clear, with no evidence of site-specific radiation damage, while Glu189 was disordered at both dose points 1 and 15, and appears to point towards solvent rather than being involved in crystal contacts. Indeed, the loop structures and density are highly similar in data set 1 and data set 15 (Supplementary Fig. S6). Notably, by this point in the dose series the majority of the images arise from crystals that began with the small cell and subsequently switched to the large cell. Other possible explanations for the dose-driven polymorph exchange are related to changes in hydration or thermodynamic factors, which could arise from heating of the microcrystal and surrounding mother liquor in the beam or from the generation of gases by radiolysis.

## 4. Conclusions and outlook   

We have demonstrated the capability to obtain room-temperature dose-dependent structures from microcrystals in silicon nitride fixed-target chips in a highly sample- and time-efficient manner. This approach is termed multiple serial structures from many crystals (MSS). An important advantage of the MSS approach is that each data set/structure within the dose series may be improved by simply repeating the measurement on additional chips and increasing the number of merged stills contributing to each dose point. This is in contrast to single-crystal experiments, in which improvements in resolution/redundancy must typically be gained at the cost of a higher dose per data set. The observed resolution of 1.48 Å is comparable to the resolution of 1.40 Å achieved using a single large room-temperature crystal at a comparable dose (Horrell *et al.*, 2018 ▸). Through the use of a serial approach, we were able to obtain serial structures at lower dose points from significantly smaller crystals using a beam with a flux density more than an order of magnitude greater.

A further advantage is that polymorphs within a batch crystal population may be separated based on unit-cell binning, leading to an improvement in data quality together with the ability to refine structures of the polymorphs independently. This is in contrast to single-crystal experiments, where polymorphs may only be visible in the form of weak additional density or dual conformations. In the future, this approach could be expanded to exploit more complex forms of grouping data, allowing polymorphs with similar unit cells to be separated, and to complement computational approaches for revealing structural heterogeneity in proteins (Lang *et al.*, 2014 ▸).

The ability to obtain dose series in a sample- and time-efficient manner at room temperature opens the door to the routine production of MSS movies of redox-enzyme function. The approach remains straightforward even when only microcrystals are available. The ability to collect data and obtain dose series using the same experimental setup as used for zero-dose SFX data collection will be particularly powerful as fine slicing of dose may allow the extrapolation of progressive X-ray-induced changes back to zero dose, which could then be verified by comparison with SFX structures determined under near-identical conditions. The approach is also well suited to time-resolved applications, as each crystal could be optically excited upon reaching the beam position and the changes then tracked over the duration of an image series. The ability, provided by a fixed-target approach, to finely control the dose and the time that each crystal spends in the X-ray beam while recording multiple slices of data is a valuable addition to the serial crystallographer’s toolbox.

## Supplementary Material

PDB reference: copper nitrite reductase, small-cell polymorph data set 1, 6gb8

PDB reference: large-cell polymorph data set 1, 6gbb

PDB reference: non-polymorph-separated data set 1, 6gby

PDB reference: large-cell polymorph data set 15, 6gcg

Supplementary Figures.. DOI: 10.1107/S2059798318010240/ba5292sup1.pdf

## Figures and Tables

**Table 1:** Data-collection, processing and refinement statistics for selected *Ac*NiR structures used for polymorph separation and dose-series data Data were either processed together or binned into small-cell and large-cell subsets prior to scaling and merging. Data were collected from a single chip (25 600 positions) with 20 sequential images each of 20 ms per position. The beam size was 8 × 8 µm, with an incident flux of 3 × 10^11^ photons s^−1^ at a wavelength of 0.9686 Å. The space group for all data was *P*2_1_3. The diffraction-weighted dose per data set was 11 kGy.

	Small cell, dose 1	Large cell, dose 1	All data, dose 1	Large cell, dose 15
Cumulative dose (kGy)	11	11	11	165
No. of integrated frames	24976	13932	38908	21836
No. of images used	23467	13481	38798	21569
Cell dimension (Å)	96.38 (0.06)	97.75 (0.05)	96.87 (0.66)	97.99 (0.14)
Resolution (Å)	29.21–1.48 (1.51–1.48)	29.21–1.48 (1.51–1.48)	29.21–1.48 (1.51–1.48)	29.54–1.80 (1.83–1.80)
*R*_merge_ (%)	87.73 (96.34)	85.70 (97.74)	94.45 (97.75)	79.52 (99.55)
*R*_split_ (%)	5.71 (87.73)	7.43 (81.12)	5.15 (54.54)	5.83 (69.07)
CC_1/2_	99.60 (72.60)	99.46 (48.66)	99.70 (55.42)	99.85 (50.16)
Mean intensity 〈*I*〉	72.3 (3.5)	65.7 (2.8)	76.8 (3.2)	28.2 (1.4)
Signal-to-noise ratio *I*/σ(*I*)	1.75 (0.17)	1.57 (0.12)	1.88 (0.15)	0.72 (0.06)
Multiplicity	548.16 (206.50)	357.91 (137.03)	927.03 (301.80)	978.36 (799.19)
Completeness (%)	100 (100)	100 (100)	100 (100)	100 (100)
No. of reflections	49882	51981	50616	29321
*R*/*R*_free_	0.186/0.216	0.205/0.227	0.235/0.276	0.167/0.205
R.m.s.d., bond lengths (Å)	0.013	0.012	0.012	0.006
R.m.s.d., bond angles (°)	1.64	1.62	1.58	0.88
Ramachandran plot				
Most favoured (%)	97.3	96.4	97.5	99.4
Allowed (%)	2.7	3.6	2.5	0.6
PDB code	6gb8	6gbb	6gby	6gcg
