# Supplementary material for: Resolving polymorphs and radiation-driven effects in microcrystals using fixed-target serial synchrotron crystallography
Source: Acta Crystallogr D Struct Biol. 2018 Nov 9;75(Pt 2):151–9. doi: 10.1107/S2059798318010240 (PMC6400251; doi:10.1107/S2059798318010240)
Supplement: Supplementary file 1 [file d-75-00151-sup1.pdf]

STRUCTURAL BIOLOGY

**Volume 74 (2018)**

**Supporting information for article:**

# Resolving polymorphs and radiation-driven effects in microcrystals using fixed-target serial synchrotron crystallography

**Ali Ebrahim, Martin V. Appleby, Danny Axford, John Beale, Tadeo Moreno-Chicano, Darren A. Sherrell, Richard W. Strange, Michael A. Hough and Robin L. Owen**

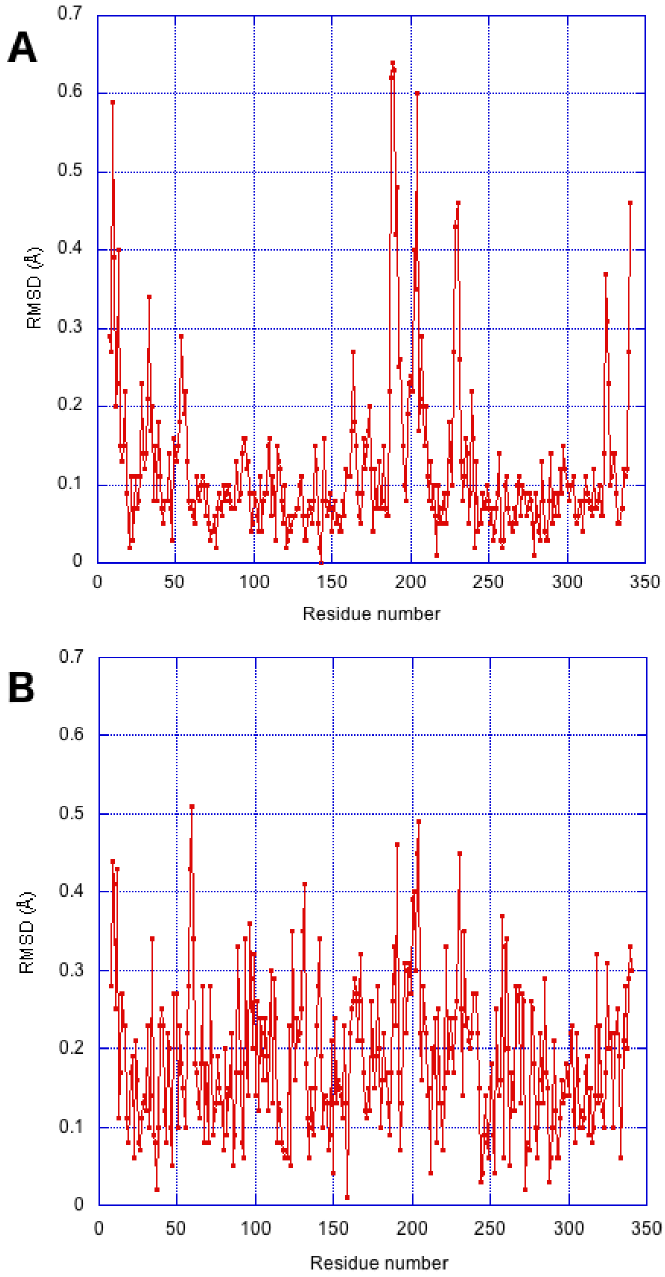


**Figure S1** All-atom RMS deviations following superposition of (A) *Ac*NiR-big and *Ac*NiR-small dataset 1 structures; (B) AcNiR-big dataset 1 and AcNiR-big dataset 15 (overall RMSD 0.21 Å). Superpositions performed in Gesamt (Kirssinel, *J. Mol. Biochem* 2012 **1(2)** 76-84)).

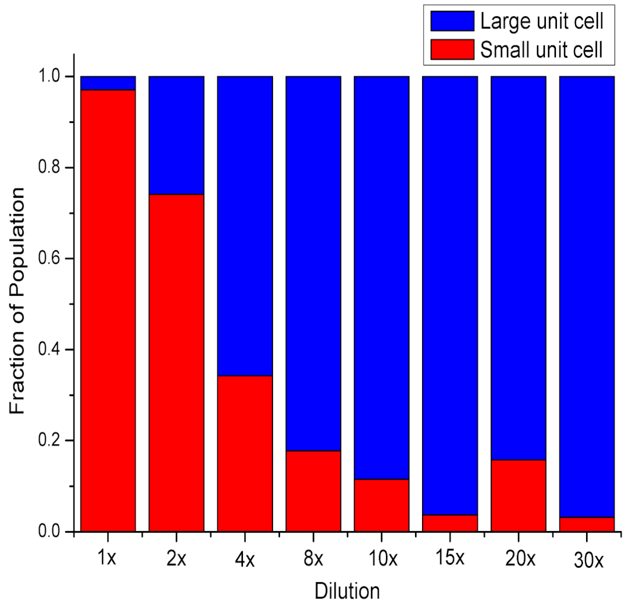


**Figure S2** Variation in fraction of small and large unit cell population as a function of microcrystal suspension concentration. Data for each concentration were collected from four city blocks (1600 positions) on a chip with the hit-rate varying from 30 to 6 %. As the microcrystal solution becomes more dilute the fraction of the small cell polymorph present decreases.

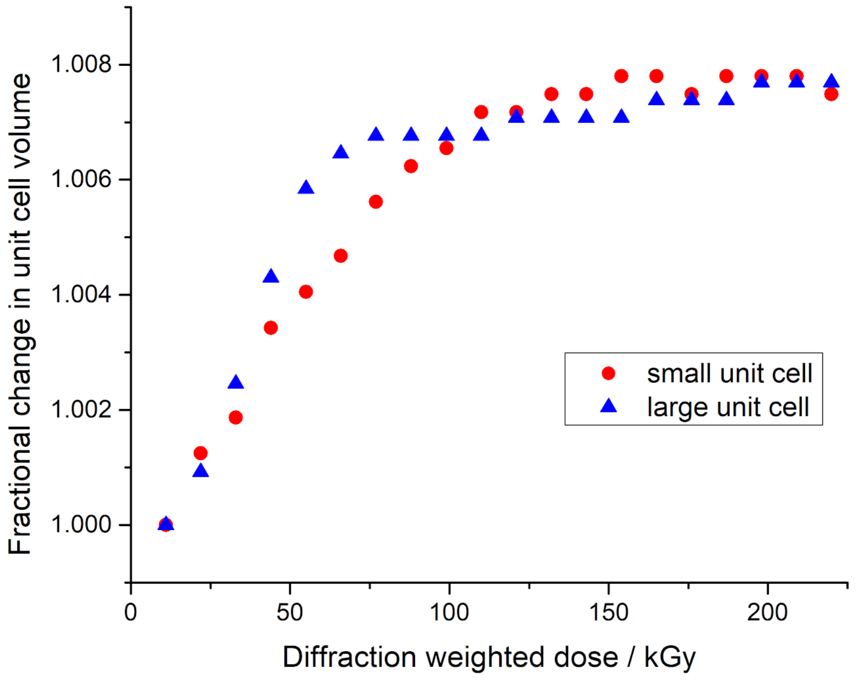


**Figure S3** Fractional change in unit cell volume for small and large cell data. It is interesting to note that the unit cell volumes change at different rates. This is in agreement with previously work (Murray & Garman 2006, JSR) suggesting that changes in unit cell volume are not a reliable metric of damage.

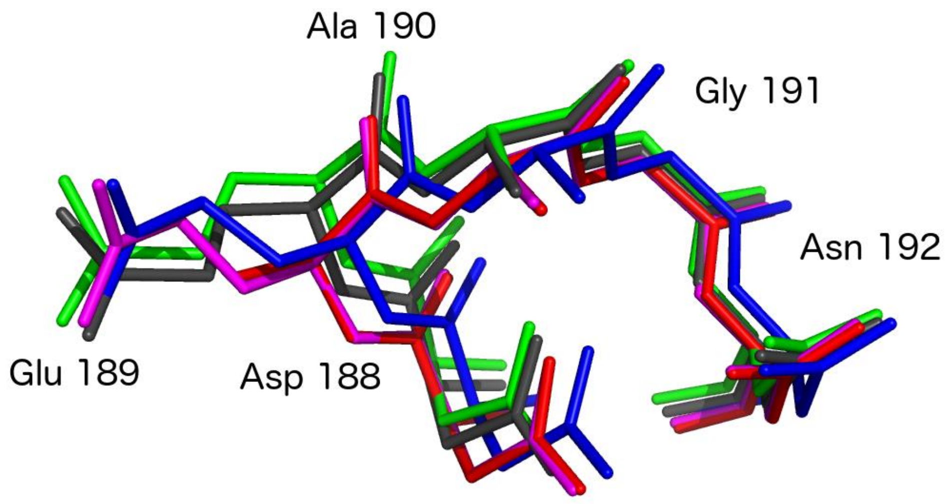


**Figure S4** Superposed loop conformations in *Ac*NiR structures determined at different temperatures and crystal sizes: red – *Ac*NiR-small polymorph (RT microcrystals), blue - *Ac*NiR-big polymorph (RT microcrystals), green - *Ac*NiR (100 K single crystal, PDB 2bw4), black - *Ac*Nir (240K single crystal, PDB 5n8f), magenta – *Ac*NiR (RT single crystal, PDB 5off).

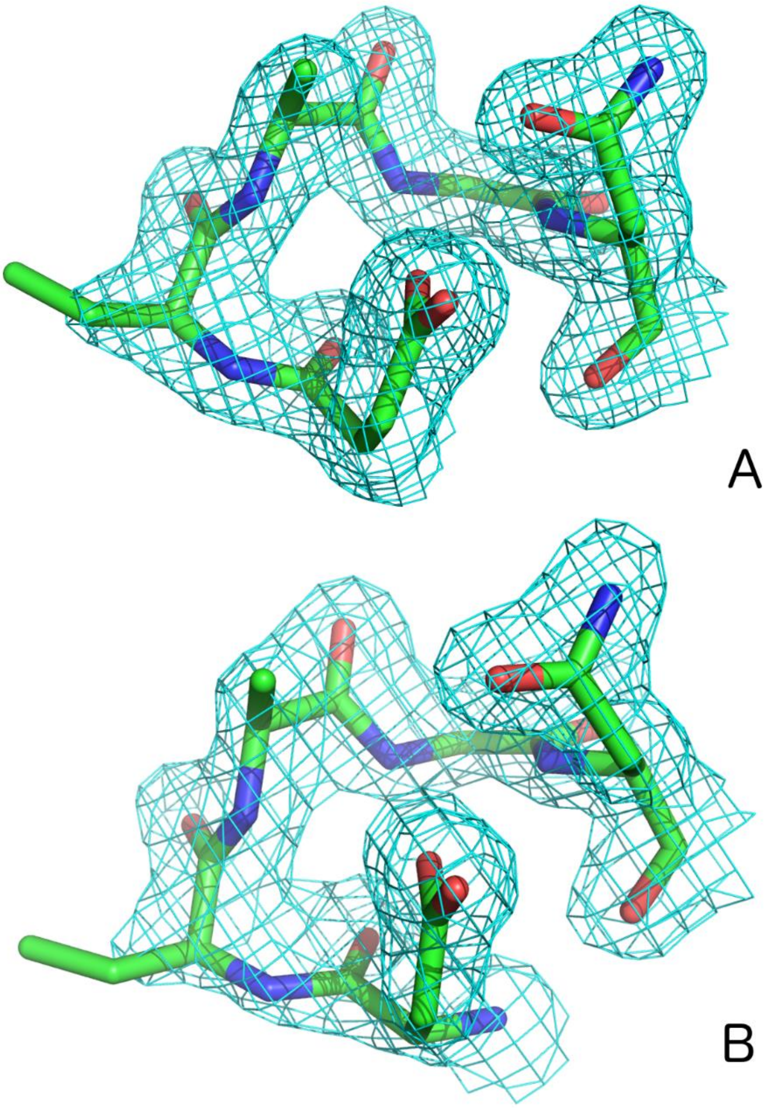


**Figure S5** Electron density maps, contoured at 1 sigma for the *Ac*NiR-big polymorph in (a) dataset 1 (dose 11 kGy, resolution 1.48 Å) and (b) dataset 15 (dose 165 kGy, resolution 1.8 Å). Note that we do not observe any negative Fo-Fc electron density peak in this region that could indicate decarboxylation of residue Asp 188 in the large cell polymorph produced after irradiation, compared to that already present in the microcrystal population at the start of the data collection.
